# Cluster detection methods applied to the Upper Cape Cod cancer data

**DOI:** 10.1186/1476-069X-4-19

**Published:** 2005-09-15

**Authors:** Al Ozonoff, Thomas Webster, Veronica Vieira, Janice Weinberg, David Ozonoff, Ann Aschengrau

**Affiliations:** 1 Department of Biostatistics, Boston University School of Public Health, 715 Albany Street, Boston, MA 02118, USA; 2 Department of Environmental Health, Boston University School of Public Health, 715 Albany Street, Boston, MA 02118, USA; 3 Department of Epidemiology, Boston University School of Public Health, 715 Albany Street, Boston, MA 02118, USA

## Abstract

**Background:**

A variety of statistical methods have been suggested to assess the degree and/or the location of spatial clustering of disease cases. However, there is relatively little in the literature devoted to comparison and critique of different methods. Most of the available comparative studies rely on simulated data rather than real data sets.

**Methods:**

We have chosen three methods currently used for examining spatial disease patterns: the *M*-statistic of Bonetti and Pagano; the Generalized Additive Model (GAM) method as applied by Webster; and Kulldorff's spatial scan statistic. We apply these statistics to analyze breast cancer data from the Upper Cape Cancer Incidence Study using three different latency assumptions.

**Results:**

The three different latency assumptions produced three different spatial patterns of cases and controls. For 20 year latency, all three methods generally concur. However, for 15 year latency and no latency assumptions, the methods produce different results when testing for global clustering.

**Conclusion:**

The comparative analyses of real data sets by different statistical methods provides insight into directions for further research. We suggest a research program designed around examining real data sets to guide focused investigation of relevant features using simulated data, for the purpose of understanding how to interpret statistical methods applied to epidemiological data with a spatial component.

## Background

Unusual geographical patterns of disease may give rise to public concern and explanations are frequently sought. Attention is often directed toward potential environmental and other factors associated with the disease in question. These investigations often have high costs in time and money, and thus it is important to verify objectively that the distribution of cases is indeed "unusual". A number of statistical methods have been suggested to assess the degree and/or the location of spatial clustering of disease cases. A good overview of the general statistical problems of clustering in the area of public health is contained in [1]. For a review with somewhat more depth but narrower scope see [2].

Despite the variety of available statistics, and the importance of understanding the methodology itself, there is relatively little in the literature devoted to comparison and critique of different methods. Most of the available comparative studies rely on simulated data ([3,4] among others) rather than real data sets. Notable exceptions include the leukemia data from upstate New York, which have been extensively analyzed with a variety of methods (see for example [5]). The advantages of using simulated data are clear, namely spatial patterns can be specified in advance and power to detect patterns under specified conditions can be considered. However, the complexity and subtleties of real data sets are frequently beyond our abilities to simulate, and the potentially large number of parameters involved in such simulations make systematic investigation of particular elements a daunting task.

In this paper we compare analytic methods using breast cancer data from the Upper Cape Cod area of Massachusetts. Geographically, the Upper Cape has interesting features that would be difficult to simulate otherwise. Its shape is roughly rectangular, but with uneven edges. Population density is highly heterogeneous, including a large non-residential "hole" in the southwest quadrant (Otis Air Force Base). These geographic features have the potential to affect various spatial methods in different ways and to different extents, making these data rich and complex in a way that simulated data often are not. We chose to compare three methods currently used for examining spatial disease patterns; one is a global test for clustering, one is a local test for clustering, and one combines a global deviance statistic with locally estimated odds ratios. All three methods are relatively simple to implement and none require commercial software. However, only the scan statistic has been implemented in stand-alone software.

We do not attempt to provide a comprehensive comparison of all available methods or to provide a complete analysis of the breast cancer data, and the reader should not interpret the results of our investigation in the context of breast cancer clusters in the Upper Cape Cod region. In contrast to the many published reports on the New York leukemia data, our purpose here is not to infer specific differences between cases and controls in the breast cancer data. Instead we aim to achieve a better understanding of the analytic properties of the methods we have selected, features of the data that may be problematic for each, and which may be most appropriate for particular situations.

It is worth noting that the three methods are not directly comparable, in the sense that one is essentially global (the M-statistic); one is local (the scan statistic); and one calculates local odds ratios along with a global deviance statistic (Webster's Generalized Additive Model (GAM)). Thus there is no reason to expect that the results of hypothesis testing using these very different methods should agree. We argue that instances where the outcome of hypothesis tests using each of these three methods are discordant may reveal important aspects of the data that could not be perceived by using any one method exclusively. In this sense, these methods provide complementary views of the data. The information contained in each approach should be considered as part of a complete and thorough investigation of spatial patterns of disease.

## Data

Data are from two population-based case-control studies of breast cancer on Upper Cape Cod, Massachusetts [6-8]. The Massachusetts Cancer Registry was used to identify incident breast cancer cases diagnosed from 1983–1993. Controls were chosen to represent the underlying population that gave rise to cases. Participants were restricted to permanent residents of the upper Cape region with complete residential histories. The case and control populations were frequency matched on age and vital status. Cases and controls were geocoded and locations entered into a Geographic Information System (GIS). For those subjects that moved during the study period, multiple residential locations were included in all analyses as appropriate.

Three latency assumptions were used in this paper. The zero latency analysis included all eligible residences i.e. exposures occurring up to diagnosis were assumed to contribute to the risk of disease. Thus all of the enrolled breast cancer cases (n = 200, representing 321 distinct residential locations) and matched controls (n = 471, representing 756 residential locations) are included in the zero latency analyses.

However, cancers initiated by exposures to environmental carcinogens may take much longer to develop. We therefore performed a 15 year and 20 year latency analysis by restricting inclusion to the residences occupied by participants at least 15 (or 20) years prior to the diagnosis (or index year, for controls). The 15 year latency analyses include 107 cases (170 locations) and 193 controls (389 locations), while the 20 year latency analyses include 248 cases (391 locations) and 341 controls (509 locations). The 20 year latency analysis includes subjects from a follow-up study, thus numbers of cases and controls are higher than would otherwise be expected due to the more restrictive latency assumption.

**Figure 1:**
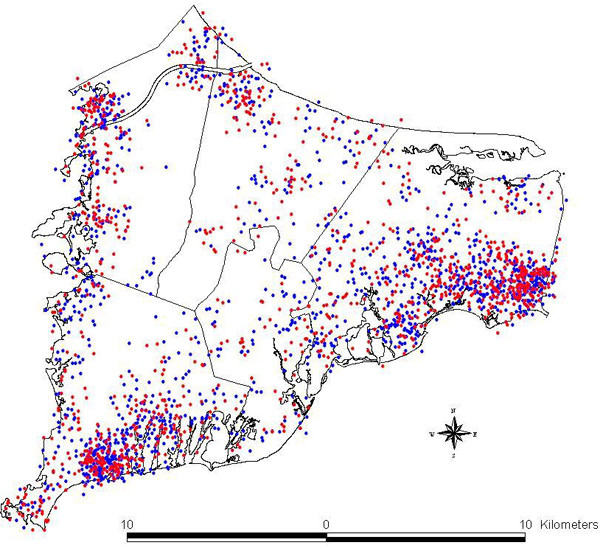
**Breast cancer cases and controls**. Distribution of breast cancer cases (in red) and controls (in blue). Each point represents the residence of one participant. Locations in this map have been geographically altered to preserve confidentiality. Actual residences were used in the analysis.

The latency assumptions thus produce three spatial patterns, giving case and control residences zero, 15, or 20 years prior to diagnosis. These data are described fully, including methodology for selection of cases and controls, demographics, and other features of the study population, in the final report of the full study as well as follow-up papers on the breast cancer data; see [7,8] for further details. For illustration, the spatial distributions of breast cancer cases and controls (with no latency assumption) are shown in Figure 1.

## Methods

The three statistical methods described here are: Bonetti and Pagano's *M*-statistic, based on the interpoint distance distribution [5]; Webster's GAM approach, which uses smoothing techniques [9]; and Kulldorff's spatial scan statistic [10]. The *M*-statistic is a global unfocused test, meaning it is only concerned with departures of the spatial distribution of cases from the distribution of controls, without determining the location of any (possibly multiple) clusters or other differences. The GAM method maps disease odds ratios, provides a global test for deviation from a flat map, and identifies locations with significantly increased or decreased risk (here GAM is the conventional designation for Generalized Additive Model, not the Geographic Analysis Machine of Openshaw [11], also used in cluster investigations). It incorporates a smoothing function for location into a conventional logistic regression which accounts for effects of covariates. Kulldorff's scan statistic, the most widely used method for cluster investigations, scans the entire study region for local excesses and/or reductions of risk. Current implementations of the binary (Bernoulli model) version of the scan statistic allow adjustment for categorical covariates only, and the *M*-statistic as implemented does not adjust for covariates at all (although allowing for categorical covariates via stratification would seem to be a straightforward extension of the existing method). For simplicity we have chosen to apply all three methods to crude data only, thus avoiding the need to consider the differences in covariate adjustment across the three methods.

### M-statistic

Bonetti-Pagano's *M*-statistic [5] is a non-parametric general test for clustering. It operates by representing and comparing the spatial distributions of two populations (here cases and controls) via the interpoint distance distribution. From any collection of *n *locations, we can calculate the roughly *n*^2^/2 interpoint distances between locations and consider the distribution of these distances. Typically, a resampling procedure on the entire study population is used to generate a baseline (or null) distribution. Both the null distribution (estimated via resampling) and the observed distribution (calculated from the interpoint distances between cases) are binned into histograms, each of which can be represented as a vector. The test statistic is then a Malhalanobis-like distance between the two vectors, weighted by an estimate of the covariance between histogram bins.

More formally, repeated resampling from the entire study population (cases and controls) is used to estimate the distribution of distances under the null hypothesis that both populations are sampled from the same spatial distribution. Binning these distances and taking the mean over all iterations gives expected counts for each bin of the histogram. Experience with this method suggests that the optimal number of bins grows roughly on the order of  where *n *is the number of cases being assessed (see also [12]). Denote by **e **the vector of expected values in each bin, expressed as a proportion of the total number of distances. Repeated resampling also allows us to estimate the covariance of **e**, which we will denote by *S*, a *k *× *k *square matrix.

The interpoint distances for the disease cases are calculated, binned, and written as a *k*-dimensional vector **o**, the observed bin values (expressed as proportions). Then the *M*-statistic is:

*M *= (**o **- **e**)'*S*^-^(**o **- **e**)

where *S*^- ^is the Moore-Penrose generalized inverse of the sample covariance matrix *S*. Thus we calculate the difference between the expected (under the null hypothesis of no clustering) bin proportions and the observed bin proportions of the disease cases, inversely weighted by the covariance estimator. As *S*^- ^is a positive semi-definite matrix, *M *≥ 0.

The asymptotic distribution of *M *is found in [5]. In practice we can use the resampling procedure to calculate the distribution of *M *empirically under the null hypothesis. Comparing the calculated value of the test statistic to the null distribution gives a p-value that can be interpreted as the probability that the spatial distribution of the disease cases differs from the entire study population by chance alone.

### GAM smoothing

Webster et al. [9,13] have used a procedure based on smoothing and generalized additive models (GAMs) to map disease and detect clusters (see [14] for related work). The generalized additive model predicts the log odds of disease (logarithm of the ratio of cases to controls) as a linear function of some covariates and a smooth function of spatial coordinates.

Specifically, the model specifies that for an individual with covariates **z_i _**and spatial location (*x*_*i*_, *y*_*i*_), the probability *p*_*i *_of disease is given by:

logit(*p*_*i*_) = *S*(*x*_*i*_, *y*_*i*_) + *β***z_i_**

where *β *denotes the vector of linear regression coefficients for the covariates. *S*(*x*, *y*) is a bivariate smooth function. Webster et al. use a loess (locally-weighted regression smoother) because it is adaptive to changes in data density typically found in population maps. Around each point in the study area, a variable sized window is constructed based on a predetermined number of nearest neighbors; within this window, the data contribute to *S*(*x*, *y*) according to a tricube weighting function. Details are covered thoroughly in [15]. The window size (span) will affect both the bias and the variance (i.e. the amount of smoothing). Reducing the span reduces the bias but also increases the variance (reducing smoothness). Various criteria have been developed to balance these two properties of the smoother. Webster et al. use the Akaike Information Criterion (AIC), which averages the deviance but penalizes the number of degrees of freedom. Minimizing the AIC estimates an "optimal" balance of bias and variance [15] in a computationally feasible manner. The global statistic tests the null hypothesis of a flat map using the deviance of the model with and without the smoothing term. Among the available global test statistics, here we have used the deviance statistic [9]. The distribution of the statistic is estimated using permutation testing, with the case-control status permuted repeatedly. A pointwise test is then used to locate areas with significantly increased or decreased log odds relative to the map as a whole (the overall case-control ratio for crude analyses). The permutations also generate a distribution of the log odds at each location under the null hypothesis. The local p-value is determined by comparing the observed log odds with the null distribution.

After all statistical tests are performed, the log odds are converted to odds ratios using the entire study population as a reference. The odds ratios are mapped and significant "hot" and "cold" spots are delineated by drawing the .025 and .975 quantiles of the pointwise p-value surface. This graphical display is a natural part of the statistic and offers a rapid interpretation of the results of the calculations. The entire procedure can be run with existing software, e.g. S-Plus for the GAM and ArcView for mapping.

We note that care should be taken when interpreting the map of local p-values, because there is no adjustment for multiple testing. Thus under the null hypothesis of identical spatial distributions of cases and controls, we can expect in general that statistically significant local p-values will occur at a higher rate than the Type I error rate specified by the nominal alpha level. In other words, the local p-values are not to be used for hypothesis testing since we do not have adequate control of the Type I error rate. The local p-values do provide information about the measure of effect (in this case the local odds ratio), but inference based on these local p-values alone should be avoided.

### Scan statistic

Kulldorff's scan statistic [10] has become the most widely used test for clustering in recent years, both because of its efficacy in detecting single hot (or cold) spots as well as the availability of the free software package SaTScan [16] for implementing the test. The basic idea of the scan statistic is to allow circular windows of various sizes to range across the study region. At each location, the rate of disease inside the window is compared to that outside the window. A hot (respectively cold) spot is characterized by a higher (lower) localized rate of disease.

In a case-control setting, the scan statistic is a likelihood ratio test statistic under a Bernoulli probability model. For a given zone (circular window) *Z *let *p*_*Z*_, *q*_*Z *_denote the probability of a data point being a case inside or outside the circle, respectively. The likelihood function under this Bernoulli model can be expressed in a straightforward fashion in terms of *p*, *q*, and the number of cases and controls inside and outside *Z*. We can then calculate:



Let  denote the zone for which *L*_*Z *_achieves its maximum. This is called the most likely cluster, and we can calculate a test statistic via a likelihood ratio test. Let *L*_0 _= sup_*p *= *q *_*L*(*Z*, *p*_*Z*_, *q*_*Z*_) be the likelihood under the null hypothesis (no clustering) and use



as the statistic of interest. The most likely cold spot is calculated similarly.

As with the other methods, inference is based on permutation of the case-control status. Under repeated permutations, the distribution of *λ *under the null hypothesis is generated, and we compare the observed value of *λ *to this distribution to yield a p-value. As noted above, SaTScan provides a relative risk for the most likely hot/cold spot, here an odds ratio inside the circle divided by an odds ratio outside the circle (hence not exactly comparable to the odds ratio computed by the GAM method).

For this study, we used the most recent version of the publicly available software [16] for analyzing binary (case-control) data, searching for either hot or cold spots.

## Results

The three statistics in question were calculated for the breast cancer data with each of three latency periods. The results, showing global p-values for the *M*-statistic and the GAM method, and local p-value (for the identified "most likely cluster") for the scan statistic, are summarized in Table 1.

**Table 1:** p-values associated with cluster statistics. Results (p-values) of analysis using the scan statistic, the *M*-statistic, and the GAM method with deviance statistic.

	Breast cancer		
	20 yr lat	15 yr lat	No lat
scan stat	0.068	0.241	0.209
*M*-stat	0.015	0.008	0.539
GAM	0.003	0.006	0.046

**Figure 2:**
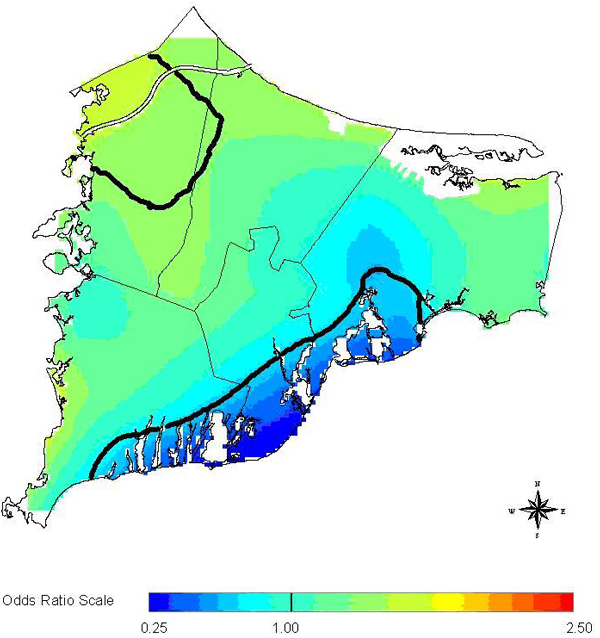
**Breast cancer 20 year latency (GAM)**. Breast cancer 20 year latency, GAM smoothed rate map. Solid lines delineate areas where the point-wise GAM deviance statistic is less than 0.05.

**Figure 3:**
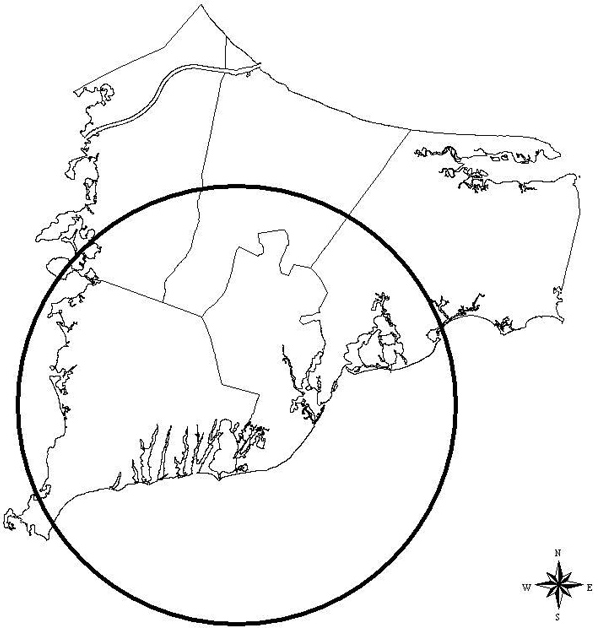
**Breast cancer 20 year latency (scan)**. Breast cancer 20 year latency, scan statistic most likely cluster. Estimated relative risk for the indicated cluster is 0.823.

**Figure 4:**
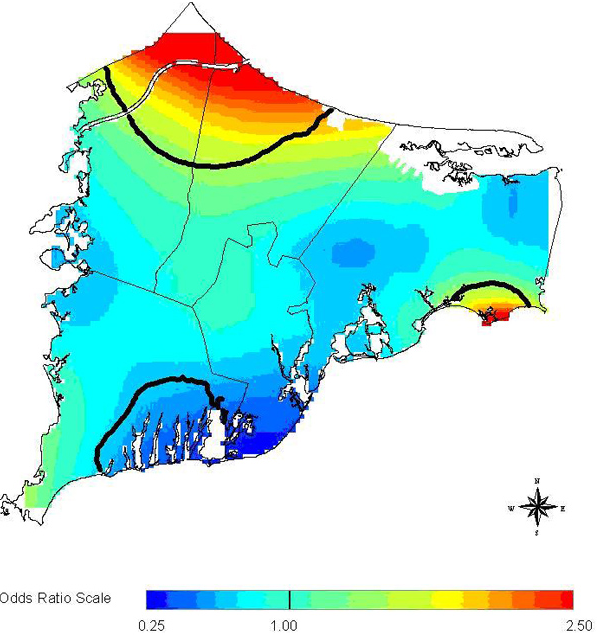
**Breast cancer 15 year latency (GAM)**. Breast cancer 15 year latency.

**Figure 5:**
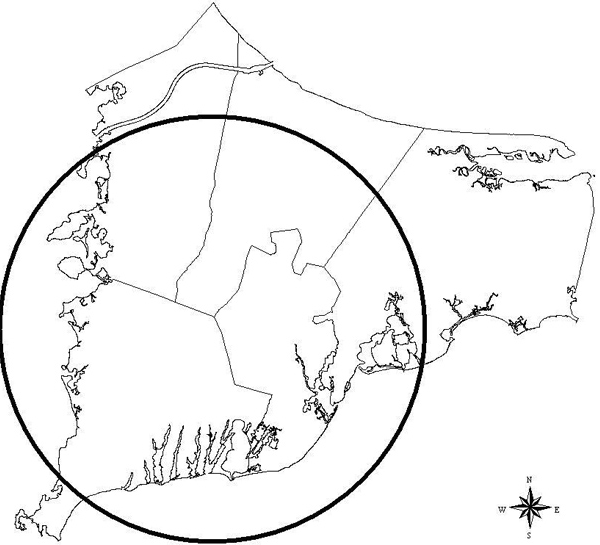
**Breast cancer 15 year latency (scan)**. Breast cancer 15 year latency. RR = 4.629.

The three methods in general are not concordant when considered in a hypothesis testing context. However, all three methods are at least suggestive of significantly different spatial patterns for cases and controls when applied to the 20 year latency data set. The scan statistic result, while not significant at the customary 0.05 level, is nonetheless indicative of an excess of cases in the calculated most likely cluster, and contributes evidence towards a difference between cases and controls when considered in the context of the results of the other two statistics. The smoothed map using the GAM method (Figure 2) shows one hot and one cold spot, a situation in which all three statistics are expected to maintain some reasonable sensitivity. The corresponding "most likely cluster" produced by the scan statistic is also shown (Figure 3). When applied to the breast cancer data set with 15 year latency, both the *M*-statistic and the GAM indicate differences in the spatial distribution of cases and controls that are very unlikely to be explained by chance. The scan statistic, however, suggests that considered locally, random variation remains a plausible explanation. Examination of the smoothed map (Figure 4) shows two distinct and prominent hot spots in the data, and one cold spot. The presence of multiple clusters in the data may partially explain the divergent results. The associated scan statistic output is also shown (Figure 5).

**Figure 6:**
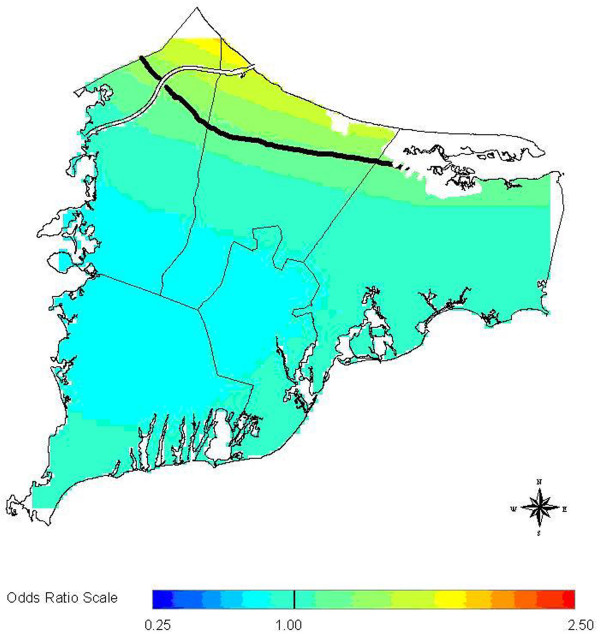
**Breast cancer no latency (GAM)**. Breast cancer no latency.

**Figure 7:**
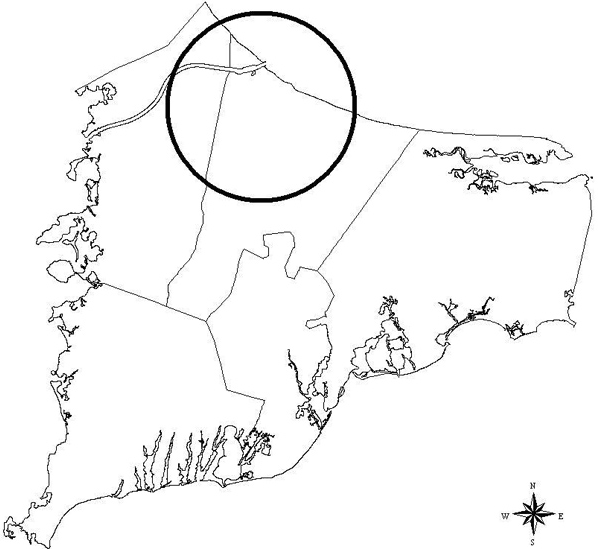
**Breast cancer no latency (scan)**. Breast cancer no latency. RR = 0.453.

When no latency is considered for breast cancer, the *M*-statistic is no longer statistically significant, making the GAM the only method that offers strong evidence against chance alone explaining the spatial patterns in the data. Figures 6 and 7 show the smoothed map for this data set as produced by the GAM and the cluster identified by the scan statistic, respectively. The GAM map shows a broad, diffuse area of increased risk (odds ratios (ORs) roughly 2.0) along the coast and periphery in the northern Cape Cod area. Kulldorff's likelihood-based method identifies the same area and roughly the same relative risk (RR), but the local excess of cases is not statistically significant. Both methods are detecting a single hot spot, but it is elongated instead of the optimal (circular) configuration for Kulldorff's method. The *M*-statistic provides no evidence of global differences at the significance level of 0.05, perhaps due to the diffuse nature of the apparent hot spot. Thus the evidence for clustering in this data set is mixed.

## Discussion

The discussion of results presented here should not be construed as epidemiologic findings, but rather the output of three statistical methods as applied to real data. The maps produced are for illustration purposes only, and should not be interpreted epidemiologically (one reason being that we have not controlled for covariates).

We remark that the common use of the word "cluster" to describe a disease hot spot represents only one kind of departure of spatial difference between cases and controls. The scan statistic alone restricts itself to this particular kind of spatial difference and further places emphasis on the single most likely circular hot or cold spot. We have chosen to adopt here the broader but more flexible objective of detecting *any *difference in the spatial distribution of cases compared to the controls. The problem of locating and quantifying local excesses or deficits is clearly important, and both the scan statistic and the GAM address this problem directly. The M-statistic does not, although extensions of distance-based methods to the problem of cluster location are currently being developed [17].

We have presented applications of three well-developed and theoretically-grounded methods to detect spatial differences in the distribution of cases and controls in a real data set. The different patterns seen in this data set, comprising breast cancer with different latency considerations, affect the outcomes of these methods. We have identified at least three features that plausibly are involved (the shape, number, and intensity of areas of inhomogeneity), but there are likely others present here and in other real data sets. For example, methods may have different sensitivity depending on the areal size and/or location of spatial differences. In these cases, the sensitivity of each method may differ depending on location of a particular hot or cold spot, even when size, shape and intensity of the hot/cold spot are comparable (e.g. differing "edge effects" across methods).

Each of these methods would be expected to have certain strengths and weaknesses. The *M*-statistic has been implemented both in case-control studies [5], and in surveillance settings [18] where there is a large amount of historical data to use as a baseline for the null distribution of distances. Simulations suggest that it has the potential to be sensitive to situations such as multiple hot spots, where other statistics (such as the scan statistic) may lose power [3,4], but these same studies show that the *M*-statistic will typically underperform other statistics when there is a single hot spot to detect.

Provided there is some historical record, or sufficiently large control population from which to resample, the *M*-statistic can handle small sample sizes adequately. This is important in a surveillance setting, and is an advantage over rate-based statistics that may have insufficient data in the small sample case to draw proper inferences. In environmental settings these situations may arise in small, neighborhood-sized population studies.

However, as currently implemented the *M*-statistic does not adjust for covariates, but instead is used on raw spatial data only. The origins of the *M*-statistic lie in public health surveillance where spatial confounders are implicitly accounted for in the immediate historical record. As noted above, the *M*-statistic does not locate hot spots, but rather detects a difference between the two populations under comparison. Because these differences are quantified via the interpoint distance distribution and not the geographic locations of cases and controls themselves, results do not have a direct interpretation as do the "most likely cluster" of the scan statistic or the local odds ratios of the GAM.

GAM smoothing is a robust data-based approach that can be run with standard software. The ability to map disease outcomes while adjusting for covariates in a way familiar to epidemiologists is a particular strength. It is semi-parametric, assuming a linear model in the covariates with an additive spatial effect. Ignoring covariates and considering the data on a purely spatial basis there are essentially no statistical assumptions required, although the choice of window size may affect the sensitivity of the smoothing approach. The GAM approach provides global statistics to test the map for overall deviation from flatness as well as a pointwise test to locate areas of significantly elevated and decreased disease risk. Sufficient sample size for stable rates is also important, and results for small sample sizes are difficult to interpret meaningfully.

The scan statistic will certainly excel [3,4] when there is a single hot spot present and that hot spot is roughly circular in shape. The model assumption of a Bernoulli distribution inside and outside a circular region can be suboptimal if either the hot/cold spot is not circular, or if there is more than one spot present. There has been additional work on the scan statistic focusing on examining or improving robustness to the shape of the hotspot [19-21].

The scan statistic is especially appealing because of its immediate identification of the most likely cluster. Public availability of the implementation via the SaTScan software has increased its popularity and visibility. Perhaps most importantly, the method's exceptional power to detect single hot spots deserves consideration in situations where a single hot spot scenario seems plausible, or even possible. As a rate-based approach, the scan statistic is also limited to sample sizes that provide stable rate estimates.

Aggregated data can be handled using a Poisson model, similar in spirit to the Bernoulli model used for case-control data. The currently available software can adjust for covariates in the Poisson case, and adjustments for categorical variables in the Bernoulli model are allowed in the most recent release of the SaTScan software.

Multiple hot/cold spots would seem to be problematic when using the scan statistic, since it uses a likelihood function from a model based on a single hot or cold spot only. Placing restrictions on the underlying probability model clearly results in higher power when the model is correctly specified, but the presence of multiple clusters would imply that the scan statistic has misspecified the model. Thus we should expect that in some of these situations the scan statistic may suffer loss of power. The GAM and M-statistic would be expected to be sensitive to a wider variety of multiple cluster arrangements, but this flexibility is inherent in the global nature of these test statistics in contrast to the essentially local nature of the scan statistic.

The published results cited above indicate that for the benchmark simulated data considered, the scan statistic is quite robust to some multiple cluster arrangements. We note that these comparisons are dependent on the simulated data used for the purposes of the power study. Multiple hot/cold spots may be common occurrences in real data sets, and a more thorough effort to generate realistic simulations for these data is a direction for future research.

Likewise, there is no reason to assume that areas of increased risk will be any particular shape, especially as neither underlying population nor possible exposures are similarly constrained. Several recent papers have continued investigation of the scan statistic and its performance when dealing with non-circular hot spots (as well as extensions of the methodology to improve robustness in these situations); see for example [17]. As with the issue of multiple hot spots, more work may be needed to simulate such data in a realistic manner. We again emphasize the importance of studies that consider real data in addition to synthetic data, and the potential to learn from both types of data as spatial methods continue to develop and improve.

## Conclusion

With the variety of approaches to the problem of examining spatial patterns of disease, it is not surprising that some methods are more effective than others for detecting certain patterns. A better understanding of the relative strengths and weaknesses of the various methods is essential to appropriate choices of methodology. Studies of spatial distribution of disease will also benefit from the information available from a variety of statistical methods, and careful consideration of the complementary nature of this information should assist in the interpretation of results of studies with a spatial component.

To this point, much of the work in gaining this understanding has come from analyzing synthetic data, where the underlying model can be controlled and various features superimposed in order to perform a careful study of these strengths and weaknesses. However, some characteristics of real data sets may be hard to simulate with synthetic data, or may not be readily apparent in advance of analysis and further study, and results based on simulated data are at least partially dependent on the particular simulations themselves.

The comparative analyses by different methods of real data sets point to directions for further research of the properties of each of the statistics used in this paper. We suggest a further research program designed around alternately examining real and simulated data sets for these kinds of differences, in order to develop the practical application of statistical methods to epidemiological data with a spatial component.

## List of abbreviations

AIC: Akaike Information Criterion

GAM: Generalized Additive Model

GIS: Geographic Information System

OR: Odds Ratio

RR: Relative Risk

## Competing interests

The author(s) declare that they have no competing interests.

## Authors' contributions

AO and VV were responsible for statistical programming. All authors contributed to writing and editing.
